# The Role of *inab* in Axon Morphology of an Identified Zebrafish Motoneuron

**DOI:** 10.1371/journal.pone.0088631

**Published:** 2014-02-12

**Authors:** Liesl Van Ryswyk, Levi Simonson, Judith S. Eisen

**Affiliations:** Institute of Neuroscience, Eugene, Oregon, United States of America; National Institutes of Health / NICHD, United States of America

## Abstract

The ability of an animal to move and to interact with its environment requires that motoneurons correctly innervate specific muscles. Although many genes that regulate motoneuron development have been identified, our understanding of motor axon branching remains incomplete. We used transcriptional expression profiling to identify potential candidate genes involved in development of zebrafish identified motoneurons. Here we focus on *inab*, an intermediate filament encoding gene dynamically expressed in a subset of motoneurons as well as in an identified interneuron. We show that *inab* is necessary for proper axon morphology of a specific motoneuron subtype.

## Introduction

For correct locomotor circuitry to form in a developing embryo, motoneuron axons must contact appropriate muscle targets. Motoneurons are classified into subtypes that are based on their axon projections and morphologies, a hallmark of appropriate motoneuron subtype specification. Correct motoneuron development is mediated by expression of genes that allow motoneurons to acquire subtype-specific characteristics, such as axon morphology. Although a number of genes have been identified with necessary roles in motoneuron development, we still have an incomplete picture of the genes required for motoneuron differentiation and axon morphology.

The zebrafish spinal cord is an ideal model in which to study questions of early neuronal development, because there are a small number of individually identifiable neurons that can be observed in live animals over the course of development [1]. The earliest-developing motoneurons in the zebrafish spinal cord are referred to as primary motoneurons (PMNs); there are also later-developing motoneurons referred to as secondary motoneurons [2]. PMNs are especially amenable to study, because they have three distinct subtypes. Each of these subtypes projects an axon to a subtype-specific region in the overlying muscle [2], [3] and expresses a number of genes differentially [4], [5], [6]. Not only can the mechanisms of PMN subtype specification be addressed genetically in zebrafish, but the genes known to be involved in zebrafish motoneuron specification are conserved across vertebrates.

To uncover additional genes involved in motoneuron development, including both differentiation and acquisition of axon morphology, we performed a microarray screen. By comparing the transcriptome of embryonic zebrafish spinal cords manipulated to have supernumerary motoneurons to that of spinal cords manipulated to have fewer motoneurons, we were able to select a number of candidate genes. These candidates were up-regulated or down-regulated in the same direction in each condition as genes already known to be expressed in zebrafish motoneurons. One of these candidate genes is *internexin neuronal intermediate filament alpha b* (*inab*) that encodes a neuronal intermediate filament. We chose to focus on this gene because of our interest in genes that potentially regulate motoneuron differentiation and axon morphology.

Neuronal intermediate filaments are a family of proteins thought to be involved in neuronal differentiation as well as acquisition of axonal morphology [7]. Upregulation of a number of neuronal intermediate filament proteins has been correlated with alterations in cellular morphology that accompany differentiation [8], [9]. The genes that encode some intermediate filament proteins, such as *nestin* and *ina*, are expressed in mammalian neuroblasts at the end of migration and beginning of differentiation [10], [11], [12], [13]. *nestin* is a well-characterized marker of neural progenitors [10], [14] and both *nestin* and *ina* are thought to be involved in differentiation of neural progenitor cells and the accompanying acquisition of proper axonal morphology [7], [9], [15], [16], [17].

The goldfish and frog *ina* homologues are required for axon outgrowth and are upregulated in retinal ganglion cell axons after optic nerve crush [18], [19], [20]. Interestingly, different intermediate filament genes are expressed in different subpopulations of neurons as they acquire distinct morphologies [9], suggesting that intermediate filaments could be important for neuronal subtype specification.

In addition to its well-characterized role in the optic nerve, *ina* is expressed in spinal motoneurons of goldfish and frog, although its role in cells other than retinal ganglion cells has not yet been elucidated [18], [21]. Zebrafish have two homologs of *ina* – *inaa* and *inab* – of which *inab* has been shown to be expressed in motoneurons [19], [22]. Its presence there raises the possibility that it could be involved in aspects of motoneuron development.

Here we investigate which zebrafish PMN subtypes express *inab*. Zebrafish have three PMN subtypes, all derived from the progenitor of motoneuron (pMN) domain: CaP/VaP, MiP, and RoP [23]. CaP and VaP are initially equivalent MNs, referred to as CaP/VaPs, that go on to acquire distinct CaP and VaP fates [24], [25], [26], [27]. CaP is present in all spinal hemisegments, and can be identified by a long, ventrally-extending axon. VaP is present in only approximately half of the spinal hemisegments, projects a short, ventrally-extending axon, and typically dies during embryonic development [24]. Both MiP and RoP are present in all spinal hemisegments. MiP can be identified by a dorsally-projecting axon, whereas RoP has a ventrally-projecting axon that arborizes more laterally than the CaP axon [2].

We also focus on an identified interneuron that arises from the pMN domain and can be a sibling to motoneurons, VeLD [23]. The VeLD cell body is located just dorsal to the PMNs [28]. VeLD can be identified by its ventral axon that descends ipsilaterally and projects caudally for many segment lengths [29], [30].

We demonstrate that *inab* is expressed only in the CaP/VaP MN subtype. Surprisingly, VeLD interneurons also express *inab*. VeLDs and PMNs are derived from the same progenitor domain, can be siblings, and have been shown to express some of the same genes [6], [23], [31]. *inab* appears unnecessary for motoneuron or interneuron specification, but the axon morphology of VaP MNs is disrupted when *inab* is misspliced – VaP axons have an increased number of processes – however the number of processes on other PMN axons is not influenced by missplicing of *inab*. This work suggests that neuronal intermediate filament proteins are involved in acquisition of axon morphology and are often differentially expressed in neuronal subtypes.

## Materials and Methods

### Zebrafish

Wild-type (AB), *smu^b641^* [32], *Tg(nrp1a:GFP)^jsl2^* [33], *Tg(mnx1:GFP)^ml2^* [34], *Tg(vsx1:GFP)* [35], and *Tg(mnx1:GAL4VP16)^b1222^*;*Tg(UAS:tdTomato:cmlc2:EGFP)^b1224^* [6] zebrafish were maintained in a laboratory breeding colony according to established protocols [36]. Embryos collected from natural crosses were allowed to develop at 28.5°C and staged according to morphological criteria and hours postfertilization (hpf) [37].

### Ethics statement

This study was carried out in strict accordance with the recommendations from the Guide for the Care and Use of Laboratory Animals of the National Institutes of Health. The protocol was approved by the University of Oregon Institutional Animal Care and Use Committee (11–20). All embryonic dissociations were performed under MS-222 anesthesia, and every effort was made to minimize suffering.

### RNA synthesis and injections

Capped dominant negative *rbpja* (formerly *Suppressor of Hairless*) RNA was synthesized and injected as previously described [38]. This was sufficient to generate ectopic motoneurons as assayed by *in situ* hybridization with *islet2a* probe [31].

Full-length *inab* RNA was transcribed using the mMessage Machine kit (Ambion/Life Technologies, Grand Island, NY, USA) according to instructions. One-cell stage embryos were injected with 400 pg *inab* RNA for rescue experiments.

### Spinal cord dissociations

Our embryonic dissociation protocol was adapted from one previously used in the lab [39]. Embryos were grown to 20 hpf and anesthetized using Finquel (MS-222) (Argent Laboratories, Redmond, WA, USA). To obtain isolated spinal cords, embryos were incubated in 7.5× pancreatin (MP Biomedicals, Irvine, CA, USA) in zebrafish physiological saline [36] until tissues began to separate (about 1 minute). Embryos were then triturated with Pasteur pipettes of decreasing size. Isolated spinal cords were transferred to Leibowitz's L15 medium (Gibco/Life Technologies, Grand Island, NY, USA) containing 10% fetal bovine serum for 1–2 minutes to inactivate the pancreatin, after which they were stored in TriReagent (Molecular Research Center, Inc., Cincinnati, OH, USA) at −80°C.

### Microarray sample preparation

Isolated spinal cords in TriReagent were pooled and homogenized. RNA extraction was performed according to standard protocols [40]. The time investment required for tissue collection prohibited analysis of biological or technical replicates. However, all genes of interest were validated as described in the Results. Extracted RNA was amplified using a MessageAmp II aRNA kit (Ambien/Life Technologies, Grand Island, NY, USA) according to supplied instructions. Amplified RNA was shipped to NimbleGen (Roche NimbleGen Inc., Madison, WI, USA) where cDNA probes were synthesized and hybridized to Zebrafish Gene Expression 385K Arrays.

### Microarray data analysis

Data analysis was carried out using ArrayStar software (DNASTAR Inc., Madison, WI, USA). Candidate genes were selected by relative transcript abundance across conditions and gene expression pattern criteria, and verified by RT-PCR and *in situ* hybridization.

### RNA probe generation

Full-length cDNA sequences containing the genes of interest were obtained from Open Biosystems (Thermo Fischer Scientific, Lafayette, CO, USA). *ccdc85al* cDNA (Clone ID: 7087329) was amplified using the following primers: forward 5′-TGTACGGAAGTGTTACTTCTGCTC-3′ and reverse 5′-GGATCCATTAACCCTCACTAAAGGGAAGGCCGCGACCTGCAGCTC-3′. *nr2f1b* cDNA (Clone ID: 6968318) was amplified using the following primers: forward 5′-AACAGCTATGACCATGATTAC-3′ and reverse 5′-GTAAAACGACGGCCAGT-3′. *inaa* cDNA was amplified using the following primers: forward 5′-CAGGTCTCAGTCTGTCTCCC-3′ and reverse 5′-TGGACAACTCCACTTCCACA-3′. *inab* cDNA (Clone ID: 7149507) was amplified using the following primers: forward 5′-TGGATAACCGTATTACCGCC-3′ and reverse 5′-CGCGCAATTAACCCTCACTAAATCACTAGTCATACCAGGATC-3′. For all genes, T3 RNA Polymerase (Roche Applied Sciences, Indianapolis, IN, USA) was used to make probe for RNA *in situ* hybridization according to standard protocols [41].

### Fluorescent RNA *in situ* hybridization

RNA *in situ* hybridization was performed according to standard protocols [41], with the following modifications: For 2-color fluorescent *in situ* hybridization, anti-sense probes were labeled with digoxygenin-UTP (Roche Applied Sciences, Indianapolis, IN, USA) and dinitrophenol-UTP (Perkin-Elmer, Waltham, MA, USA). Following overnight hybridization, unbound probe was removed with three 30-minute washes at 67°C in 50% formamide, 5× SSC, and 0.1% SDS, followed by stringent washes in 50% formamide, 2× SSC, and 0.1% Tween-20. Labeled probes were detected with HRP-conjugated anti-DIG (1∶1000; Jackson ImmunoResearch, West Grove, PA, USA) or HRP-conjugated anti-DNP (1∶1000; Perkin-Elmer), and stained with fluorescein or Cy-3-tyramide (1∶50; Perkin-Elmer) for 1-10 minutes.

Probes used include *islet1* and *islet2a* [31]; *gad1b* and *gad2* (collectively referred to as *gad*), *slc17a6a*, *slc17a6b*, and *slc17a7* (collectively referred to as *vglut*), *slc6a9* and *slc6a5* (collectively referred to as *glyt*) [42]; *vsx2* [43]; and *ccdc85al*, *nr2f1b*, *inaa*, and *inab*.

### Immunohistochemistry

Embryos were fixed overnight in 4% paraformaldehyde and 1x Fix Buffer at 4°C. Embryos were then blocked in 5% normal goat serum, 2.5% DMSO, and 0.1% Tween-20 in 1× PBS before overnight incubation in diluted primary antibody at 4°C. Unbound primary antibodies were removed by washing for 2 hours in 1× PBS plus 0.1% Tween-20, followed by overnight incubation in diluted secondary antibody at 4°C.

Antibodies used include monoclonal mouse anti-GFP 3E6 (1∶1000; A11120, Molecular Probes/Life Technologies, Eugene, OR, USA); rabbit polyclonal anti-Mnx1 and anti-Mnx2b [6]; rabbit polyclonal anti-Mnx1 (1∶1000), anti-Mnx2a (1∶1000), and anti-Mnx2b (1∶1000; AnaSpec, Fremont, CA, USA); and Alexa-Fluor dye-labeled secondary antibodies (Molecular Probes/Life Technologies).

### Morpholino injections

**Figure 1:**
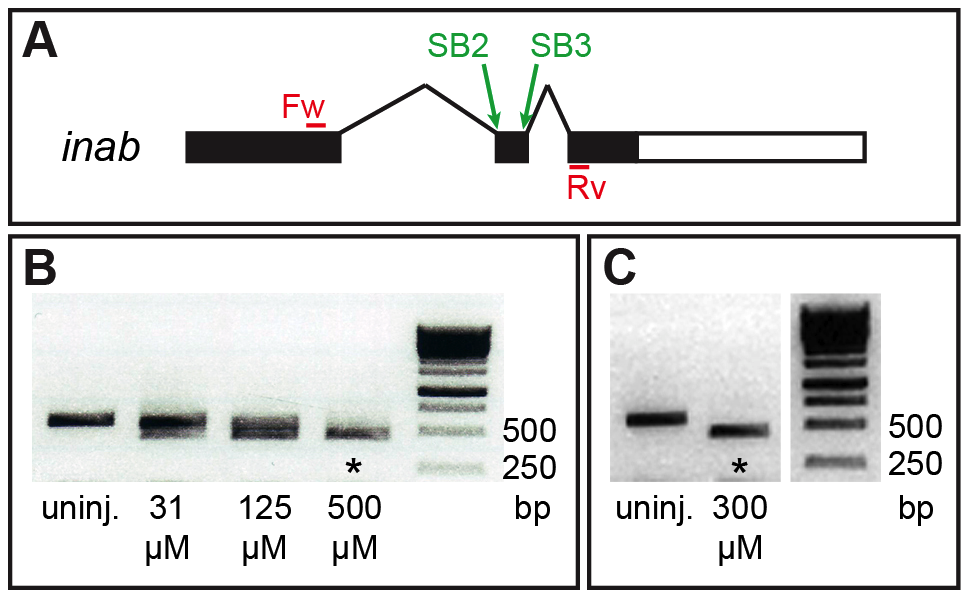
Splice-blocking MOs effectively disrupt *inab*. (**A**) Schematic of the *inab* gene. *inab* has three exons and two introns. Splice-blocking (SB) MOs target various splice sites in the gene. SB2 targets the intron1/exon2 boundary, and SB3 targets the exon2/intron2 boundary. Forward (Fw) and reverse (Rv) primers sit in the first and third exons, respectively. (**B**) PCR results confirming that uninjected embryos have wild-type *inab* (584 bp band), embryos injected with 2 nL of a combination of 31 µM SB2 and 31 µM SB3 MOs, or 2 nL of a combination of 125 µM SB2 and 125 µM SB3 MOs have both wild-type and misspliced *inab*, and embryos injected with 2 nL of a combination of 500 µM SB2 and 500 µM SB3 MOs have only misspliced *inab* (asterisk, 459 bp band). (C) PCR results confirming that uninjected embryos have wild-type *inab* (584 bp band), and embryos injected with 2 nL of a combination of 300 µM SB2 and 300 µM SB3 also have only misspliced *inab* (asterisk, 459 bp band).

Approximately 2 nL of 300 µM splice-blocking (SB) morpholinos (MOs) (Gene Tools, LLC, Philomath, OR, USA) against *inab* (a combination of 300 µM SB2: 5′-GGAATCCTAGATGACGTGATAATTC-3′ and 300 µM SB3: 5′-CAGTGATGGTTTATTACCTGTAAGC-3′) were injected into 1 to 2-cell stage embryos. This was sufficient to cause complete missplicing as assayed by primers designed to flank the splice sites (forward: 5′-CCTGGAGAAAAAGGTCGAATCC-3′ and reverse:

5′-GCTATTTTCTATGTCAA GCGCC-3′) (Figure 1B–C). Using these primers, the wild-type band is 584 bp and the SB MO band is 459 bp, indicating a complete removal of the 125 bp second exon. An additional splice-blocking MO against *inab*, SB1 (5′-ATGAAAACTGGAAAACCAACCTGGT-3′) was tested, although it did not cause complete missplicing at any concentration and thus was not used in this study.

Other previously validated morpholinos used in this study include: 2.5 nL of 100 µM *mnx1* (5′-ACCTCACAAACAGATTAACGCCTCG-3′), *mnx2a* (5′-ACCTCACAAACAGATTAACGCCTCG-3′), and *mnx2b* (5′-GACTTTTCCATTGCAACACTTTTGT-3′) to knock down *mnx* genes [6]. Morpholino effectiveness was verified by whole-mount immunohistochemistry using anti-Mnx1, anti-Mnx2a, and anti-Mnx2b antibodies (see Immunohistochemistry).

### DNA Injections

To visualize individual neurons in the trunk, approximately 3 nL of 10 ng/µL *UAS:EGFP^CAAX^:pA^395^* plasmid (prepared using multisite Gateway technology [44]) was injected into 1 to 2-cell stage *Tg(mnx1:GAL4)* embryos. We selected embryos with GFP-expressing cells for immunohistochemistry.

### Image acquisition

All images were acquired on a Zeiss Pascal confocal microscope (Carl Zeiss Microscopy, LLC, Thornwood, New York, USA) using either a 20× objective or a 40× water-immersion objective. The brightness and contrast of images was adjusted using Photoshop CS5 (Version 12.0, Adobe Systems, Inc., San Jose, CA, USA).

### Image Quantification

All observations of PMNs were made in mid-trunk spinal cord adjacent to somites 8–12.

Image analysis was performed using a previously described pipeline [45]. First, we used the NeuronJ plugin [46] (NeuronJ: http://www.imagescience.org/meijering/software/neuronj/) for ImageJ (NIH, Bethesda, MD; ImageJ: http://rsb.info.nih.gov/ij/) to define the positions of neurites within raw confocal images. We used this neurite location data and the NeuronStudio software [47] (NeuronStudio: http://research.mssm.edu/cnic/tools-ns.html) to construct a connectivity diagram of each neuron, such that each branching neurite may only give rise to two daughter neurites. We examined these data, corresponding to the complete two-dimensional arbor of each cell, using the MATLAB-based automated Sholl analysis program - Bonfire [45]. Sholl analysis produces a matrix of values corresponding to neurite length, number of branch points, and number of terminal points, as a function of radial distance from the soma. Images were scored blind, and resulting data were transferred to Excel for statistical analysis. Significance was determined using a two-tailed Student's T-test, with a p-value of less than 0.05 being considered significant. We also performed a noncentral t-distribution cumulative distribution function test which provides the minimum total sample size necessary to determine whether two samples are different from one another [48]. According to this test, a sample size of 10 provides a reliable measure for our data. We examined 14 VaPs, 11 CaPs with VaPs, 11 CaPs without VaPs, and 10 MiPs.

## Results

### Expression profiling of zebrafish spinal cord using NimbleGen microarrays

We performed a microarray screen to uncover additional genes that are expressed in zebrafish PMNs and thus potentially involved in motoneuron development. For the screen, we compared spinal cords of embryonic zebrafish with supernumerary motoneurons to spinal cords with fewer motoneurons. To create embryos with more motoneurons than normal, we injected wild-type fish with mRNA encoding a dominant negative Rbpja protein (*dnrbpja*). This dominant negative protein interferes with Delta/Notch signaling in the embryo, thus creating supernumerary early-born neurons and increasing the number of PMNs [38]. On average, the number of *islet2* ^+^ PMNs per hemisegment increases from 1–2 in control embryos to 3–6 in *dnrbpja* RNA-injected embryos [49]. To create embryos with fewer than normal motoneurons, we incrossed fish of the *smoothened* mutant line, *smu^b641^*. The Smoothened protein plays a crucial role in Hedgehog signaling, which is required for normal motoneuron development [50]. Homozygous *smu^b641^* mutants have disrupted Hedgehog signaling and consequently almost completely lack motoneurons, having on average 18 *islet2* ^+^ PMNs per embryo [32], [50].

To cut down on background from other tissues, we separated spinal cords from the rest of the tissues in the 20 hpf embryonic fish using our previous dissociation procedure [39]. After collecting a sufficient number of spinal cords (∼200 per condition), we extracted RNA from each sample and amplified it. To confirm that spinal cords were present in each case, we used tissue-specific primers to verify presence of spinal cord genes and absence of contaminating hindbrain and muscle genes (Figure S1). The amplified RNA was delivered to NimbleGen, where it was hybridized to microarrays.

We compared 10 genes known to be involved in motoneuron development – *islet1*, *nkx6.1*, *nkx6.2*, *mnx1*, *mnx2b*, *met*, *scn8aa*, *dla*, *neurog1*, and *nfasca* – and found that they were approximately 1.5 times upregulated in the supernumerary motoneuron condition as compared to uninjected, wild-type control embryos, as well as approximately 1.5 times downregulated in the decreased motoneuron condition as compared to wild-type control embryos. We then selected genes that were at least 1.5 times upregulated in the supernumerary motoneuron condition and 1.5 times downregulated in the decreased motoneuron condition, giving a list of 740 candidates.

We narrowed the list down by excluding “housekeeping” and hypothetical genes, giving a list of 101 candidate genes (Table S1). After excluding those genes expressed outside of the central nervous system and those initially expressed earlier than the birth of MNs or significantly after their maturation, we ended up with a list of 11 candidate genes (Table S1). To verify and further investigate expression patterns of the candidate genes, we obtained a clone of each gene from Open Biosystems and synthesized RNA probes for each of them. Here we provide information on three candidate genes that are expressed in ventral spinal cord.

One of the candidate genes, *ccdc85al*, is predicted to encode a transcription factor, and although it is expressed in the spinal cord, it is not expressed in PMNs and was therefore excluded from our analysis (Figure S2). Another candidate, *nr2f1b*, is a transcription factor-encoding gene that is expressed in MNs, although it is also broadly expressed throughout the spinal cord (Figure S2). Because its expression pattern is so broad, we excluded *nr2f1b* from our analysis as well. Here we describe expression and functional analysis of the third candidate gene, the intermediate filament-encoding gene *inab*, which we found to be expressed specifically in PMNs.

### *inab* is dynamically expressed in a subset of primary motoneurons and in VeLD interneurons

**Figure 2:**
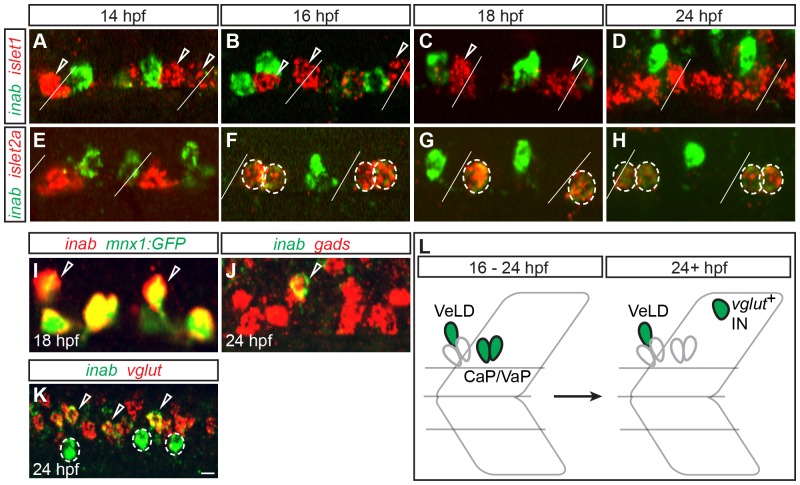
*inab* is dynamically expressed in PMNs and INs. (**A–K**) Single confocal slices of embryos labeled with *inab*, *islet*, and neurotransmitter riboprobes (*gads* for GABA and *vglut* for glutamate). Diagonal lines represent somite boundaries. At 14 hpf, *inab* is coexpressed with neither *islet1* (A) nor *islet2a* (E). Between 16 hpf and 24 hpf, *inab* is expressed in *islet2a* ^+^ PMNs (circled cells in F–H) but not *islet1* ^+^ PMNs (arrowheads in A–C). *inab* is expressed in the VeLD IN, as determined by coexpression with both GFP in *mnx1*:*GFP* transgenic embryos (arrowheads, I) and *gad* mRNA (arrowhead, J). At 24 hpf, *inab* is coexpressed with *vglut* (arrowheads, K) in a cell dorsal to the VeLD IN (circle, K). (**L**) Schematic of *inab* mRNA dynamics during early development. Between 16–24 hpf, *inab* is expressed in both CaP and VaP MNs and in VeLD INs. After 24 hpf, *inab* expression in CaP and VaP is downregulated, although it persists in VeLD and an additional, dorsally-located, glutamatergic IN. Scale bar, 5 µm in A–J; 10 µm in K.

We characterized expression of *inab* in the zebrafish spinal cord using RNA *in situ* hybridization. To determine whether PMNs express *inab*, we labeled embryos for *islet1* mRNA, which all PMNs express before 14 hpf [31], and *inab*. In embryos older than 14 hpf, we labeled with probes for both *islet1* mRNA (which after 14 hpf is expressed in PMN subtypes MiP and RoP) and *inab*, or *islet2a* mRNA (which after 14 hpf is expressed in PMN subtype CaP/VaP) [31] and *inab* (Figure 2A–H). *inab* mRNA was not detected before 14 hpf. *inab* is expressed in CaP and VaP between 16 and 24 hpf (Figure 2F–H) and is subsequently downregulated in these cells. We never saw coexpression of *inab* and *islet1*, indicating that *inab* is not expressed in either MiP or RoP.

**Figure 3:**
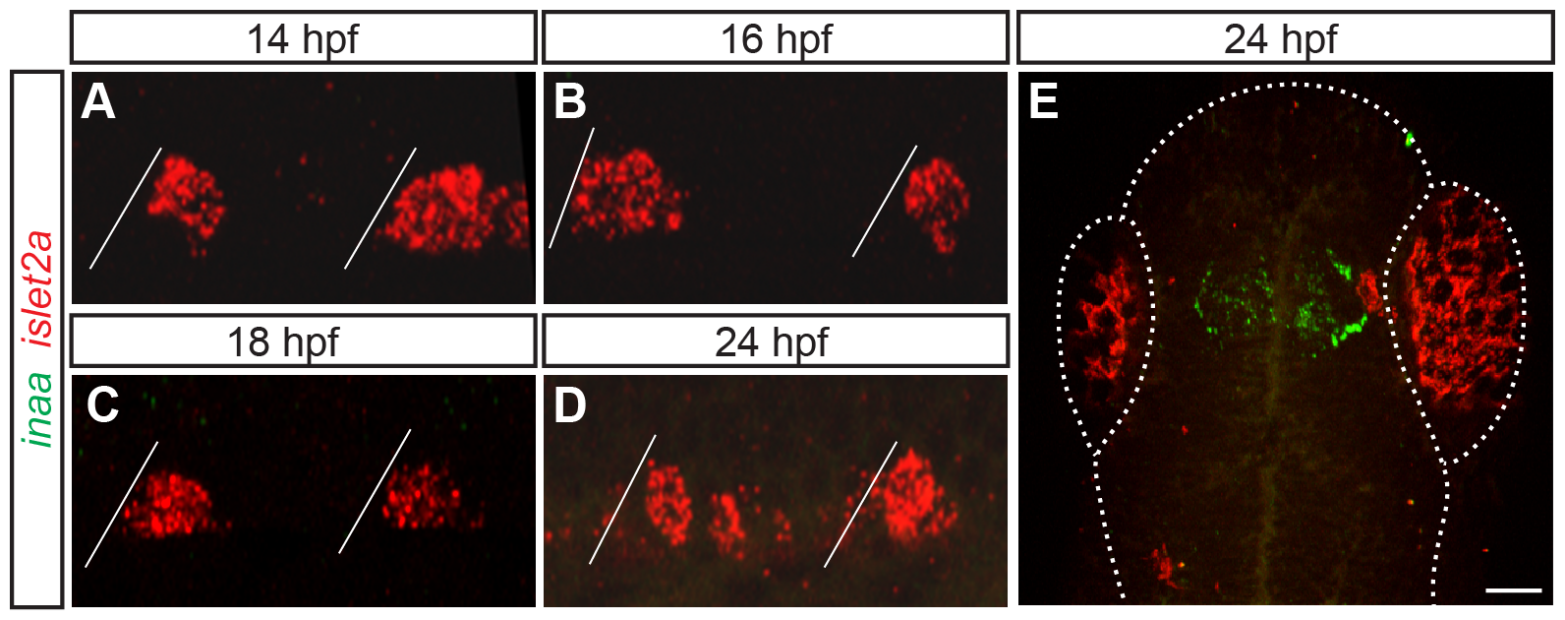
*inaa* is expressed in the zebrafish head and not in PMNs. (**A–E**) Z-projections of confocal stacks of embryos labeled with *inaa* and *islet2a* riboprobes. Diagonal lines represent somite boundaries. Between 14 hpf and 24 hpf, *inaa* is never coexpressed with *islet2a* in the trunk (A–D). At 24 hpf, inaa expression can be seen in a region of the head that corresponds to the approximate location of the diencephalic ventricle (E). Boundaries of the fish head and eyes are marked with a dotted line. Scale bar 10 µm in A–D; 50 µm in E.

In addition to its expression in a subset of PMNs, *inab* is also expressed in cells that are more dorsal than the PMNs. To determine the identity of these cells, we labeled transgenic *mnx1*:GFP embryos with *inab* riboprobe. The *mnx1*:GFP transgene is expressed in all PMN subtypes and in VeLD interneurons (INs) before 24 hpf [6]. VeLD can also be identified by expression of the neurotransmitter GABA [6], [51], [52]. The *inab* ^+^ IN expresses both the *mnx1* transgene and is labeled by an mRNA probe against *gads*, that encode the GABA synthetic enzymes, leading us to conclude that it is VeLD (Figure 3I–J). *inab* expression in VeLD is initiated at 14 hpf and persists through 24 hpf.

*inab* is also expressed in a more dorsal, glutamatergic population of interneurons, with expression initiating around 18 hpf (Figure 2K). Because we are interested in genes regulating motoneuron development, we excluded these neurons from our analysis.

There is a second zebrafish gene, called *inaa*, that is closely related to *inab* (http://zfin.org/action/marker/view/ZDB-GENE-060531-65). In the zebrafish genome, *inaa* is located on chromosome Dre13, whereas *inab* is found on chromosome Dre1. Teleost orthologs of *inaa* and *inab* are found on chromosomes Ola15 and Ola1 in medaka, on Tetraodon chromosomes Tni17 and Tni18, and in stickleback on chromosomes GacVI and GacIX, respectively. All these pairs of chromosomes contain chromosomal blocks duplicated during the course of the teleost genome duplication (TGD) derived from ancestral pre-TGC chromosome *d*. This strongly suggests that *inaa* and *inab* are paralogs of each other generated during the TGD as well [53], [54]. We characterized expression of *inaa* using RNA *in situ* hybridization. We found that *inaa* is not expressed in the spinal cord between 14–24 hpf (Figure 3 A–D), and instead is expressed in only the diencephalon or adjacent to the diencephalic ventricle at 24 hpf (Figure 3E). We therefore excluded *inaa* from our analysis.

### *inab* is expressed in hybrid cells generated by *mnx* gene knockdown

**Figure 4:**
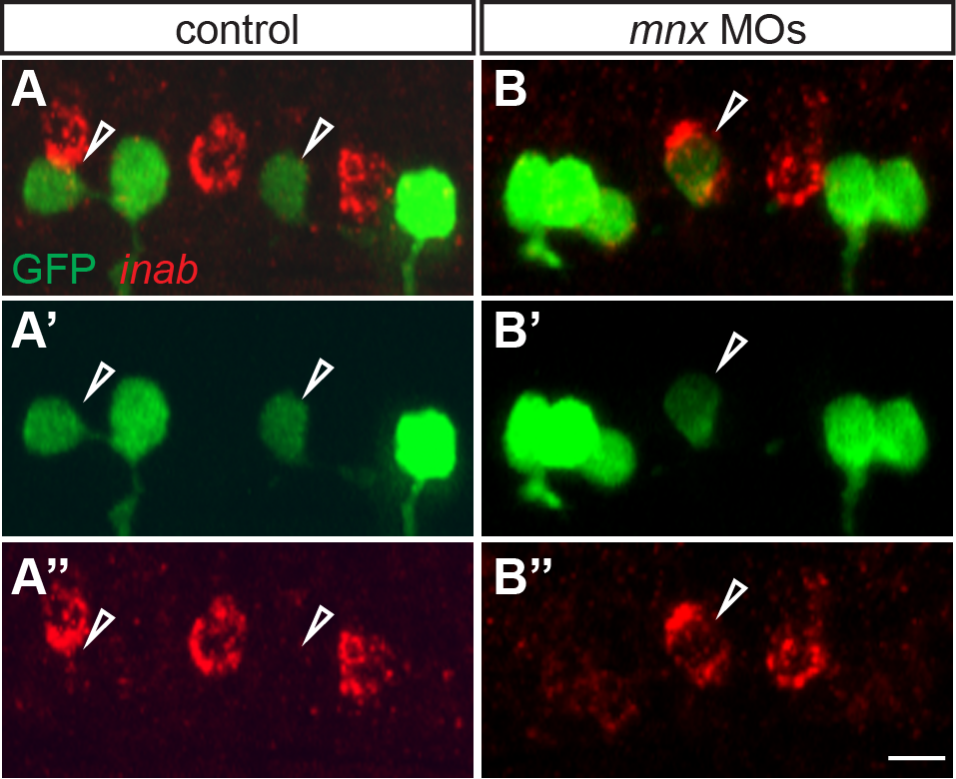
*inab* is expressed in MiP-CaP hybrids generated by Mnx protein knockdown. (**A–B**) Z-projections of confocal stacks of control and Mnx MO-injected *nrp1a:GFP* embryos labeled with *inab* riboprobe and anti-GFP antibody. At 26 hpf, *inab* is not expressed in GFP^+^ MiP MNs in control embryos (arrowheads, A-A″), but is expressed in a small percentage of GFP^+^ MiP MNs in MO-injected embryos (arrowhead, B-B″). Scale bar, 10 µm.

We recently showed that in the absence of the three zebrafish Mnx homologs (Mnx1, Mnx2a, and Mnx2b), some MiP motoneurons become MiP-CaP hybrids that develop CaP-like axons [6]. MiPs do not normally express *inab* (Figure 2), but we hypothesized that some MiPs would express *inab* in the absence of Mnx proteins. To test this hypothesis, we injected previously-validated [6], translation-blocking MOs against all three *mnx* genes into embryos of the *Tg(nrp1a:GFP)* line, in which MiPs weakly express GFP after 20 hpf. In MO-injected embryos, 32% of GFP^+^ MiPs expressed *inab* (n = 16/50 MiPs, 10 embryos; Figure 4B), in contrast to 6% of GFP^+^ MiPs in control embryos (n = 3/50 MiPs, 10 embryos; Figure 4A). This indicates that *inab* expression can be acquired by MiP-CaP hybrids that are generated in the absence of Mnx proteins, providing another molecular marker of CaP fate.

### *inab* is required for proper morphology of VaP axons

To test the function of *inab* in PMN development, we used splice-blocking (SB) MOs to prevent proper splicing of *inab* mRNA. We designed MOs to target the exon 1/intron 1 boundary (SB1), the intron 1/exon 2 boundary (SB2), and the exon 2/intron 2 boundary (SB3) (Figure 1A). Injection of up to 2 nL of either 900 µM SB1, 900 µM SB2, or 900 µM SB3 alone decreased the amount of correctly-spliced *inab* (data not shown). Only co-injection of 2 nL of 300 µM SB2 and 300 µM SB3 resulted in missplicing of all *inab* transcript (Figure 1B), although we do not know whether missplicing creates a non-functional Inab protein. What follows is an analysis of the result of missplicing of *inab* caused by co-injection of 2 nL of 300 µM SB2 and SB3 MOs.

*inab* is expressed in CaP, VaP and VeLD, so we hypothesized that its knockdown would have an effect on the axons of these particular cells. To assay any changes in axon morphology, we co-injected SB2 and SB3 MOs into *nrp1a:GFP* transgenic embryos, in which GFP is expressed exclusively in CaP/VaPs beginning at 18 hpf [33], and *mnx1:GFP* transgenic embryos, in which GFP is expressed in all PMNs and VeLD from 14 hpf on [6].

CaP/VaPs begin projecting axons ventrally around 16–17 hpf [2], [3]. These axons are obvious by 20 hpf in both *mnx1:GFP* and *nrp1a:GFP* transgenic embryos [33], [34], [55]. VeLD also extends its axon posteriorly before 18 hpf, and it too can be clearly identified in *mnx1:GFP* transgenic embryos by 20 hpf [6]. In control embryos, CaP, VaP and VeLD axons are present at 22 hpf. In MO-injected embryos, CaP, VaP and VeLD axons are also present and have a similar overall morphology to those of control embryos (Figure S3A–B). However, the ventral motor nerve, which contains axons of both CaP and VaP MNs appeared to have more small processes than ventral motor nerves in control MO-injected embryos (Figure S3A–B), suggesting a role for *inab* in axon branching or filopodia formation.

**Figure 5:**
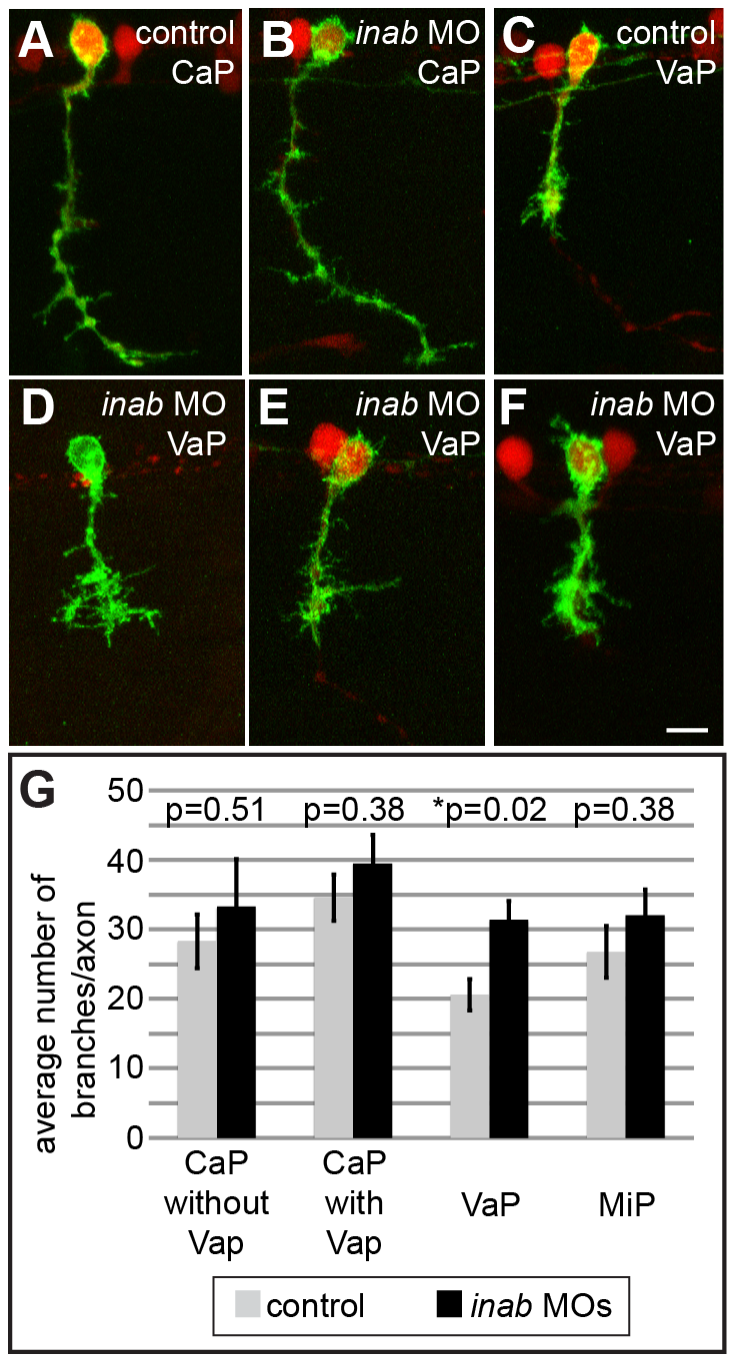
*inab* is required for morphology of VaP axons. (**A–F**) Z-projections of confocal images of control and *inab* MO-injected embryos. At 26 hpf, *UAS:EGFP* plasmid injection into *mnx1:GAL4;UAS:tdTomato* transgenic embryos reveals similar overall morphology of CaP neurons in both control (A) and MO-injected embryos (B). VaP axons in MO-injected embryos (D–F) have more processes than VaP axons in control embryos (C). (**G**) Graph representing average number of branch points per axon in individually-labeled cells in control and MO-injected embryos. No significant difference between conditions for CaP neurons alone (n = 11, p = 0.51), CaP MNs that are located next to VaP MNs (n = 11, p = 0.38), or MiP MNs (n = 10, p = 0.38). Only the increase in VaP axon processes is significant (n = 14, p = 0.02). Scale bar is 10 µm in A–F.

To learn which axons in the ventral nerve had extra processes, we injected *UAS:EGFP* plasmid into *mnx1:GAL4* transgenic embryos, which allowed us to visualize individual CaP and VaP axons, and then used Bonfire analysis to examine the patterns of processes extending from individual CaP and VaP axons in control and MO-injected embryos. The Bonfire program uses spatial information about the position of neurites to quantify characteristics such as the number of branch points along the length of an axon, or the relative connectivity of the neurites [45]. We found no significant difference in the number of processes of CaP axons between control and MO-injected embryos, irrespective of whether CaP was adjacent to VaP (Figure 5A–B, 5G). We also saw no difference in the number of processes along VeLD axons (data not shown). Another PMN, MiP does not express *inab* (data not shown), thus we used MiP as a control to examine specificity of the axon process phenotype of VaP. We saw no difference in processes along control and MO-injected MiP axons (data not shown). We did not examine RoP, another PMN that does not express *inab* (Figure 2), because RoP axons are often difficult to visualize until much later in development [2]. In contrast, VaP axons in *inab* MO-injected embryos had more processes than control VaP axons (n = 14, p = 0.02, Figure 5C–F, 5G), a phenotype that was rescued by coinjection of *inab* MO and full-length *inab* mRNA (n = 13, p = 0.14). We also examined the localization of these supernumerary processes and determined that they were significantly different along the VaP axon shaft (p = 0.017), whereas there was not a significant difference along the VaP growth cone (p = 0.20).The small but significant difference in the number of processes along the VaP axon shaft indicates that while the overall morphology of ventral motor axons is unchanged by *inab* knockdown, process formation along the length of the VaP axon is increased. Surprisingly, injection of *inab* MOs has little other effect on VaP cell fate – VaPs in MO-injected embryos still typically died by 36 hpf as shown in previous studies of control VaPs [24], [25] (data not shown).

These results demonstrate that the phenotype of *inab* knockdown is surprisingly specific to a single *inab*-positive primary motoneuron in the ventral spinal cord.

## Discussion

### Microarray candidate genes are expressed in the zebrafish spinal cord

We used microarrays to identify genes that are expressed in zebrafish motoneurons and therefore potentially involved in motoneuron development. In our microarrays, we compared transcripts from spinal cords of embryos lacking Sonic hedgehog signaling to spinal cords from embryos lacking Notch signaling. We have previously shown that both of these signaling pathways affect PMNs – excess PMNs are present in *dnrbpj*-injected embryos [38] and PMNs are decreased in *smu* mutant embryos [50]. However, both signaling pathways also affect other cell types in the ventral spinal cord [1]. Thus, although we specifically focused our attention on PMNs, some of the genes identified in our microarray may be expressed, and regulated similarly to PMN-specific genes, but in other ventral spinal cord cell types.

We identified three candidate genes that are expressed in the zebrafish ventral spinal cord. Here we show that one of these candidates, the predicted transcription factor-encoding gene *ccdc85al*, is expressed in a population of cells just dorsal to the PMNs. Although it is not expressed in motoneurons, it may be expressed in closely-related interneurons that are generated from the same progenitor domain as motoneurons in zebrafish [23]. Investigation of *ccdc85al* could potentially be interesting, because much remains to be learned about interneurons that are generated from the zebrafish pMN domain.

We also show that another candidate gene from the microarray, the transcription factor-encoding gene *nr2f1b*, is expressed broadly throughout the zebrafish spinal cord, including expression in PMNs. Based on its expression pattern, this gene may be less informative about mechanisms of motoneuron development than a gene with a cell type-specific expression pattern. The presence of *nr2f1b* in the spinal cord, however, merits further investigation.

### Zebrafish have two *ina* paralogs

We show that the neuronal intermediate filament-encoding gene *inab* is expressed in a subset of zebrafish PMNs. In addition to *inab*, zebrafish also have another paralog of *ina*, named *inaa*, which we investigated alongside *inab*. We found that *inaa* is dynamically expressed in embryonic zebrafish, although not in PMNs during early development. This suggests that *inaa* and *inab* have diverged in function, leaving only *inab* with a role in PMN development.

### *inab* is dynamically expressed in identified zebrafish spinal neurons

We found that *inab* is expressed in a subset of zebrafish PMNs – CaP and VaP – and a closely-related interneuron – VeLD. Previous studies of genes involved in PMN specification have underscored a close relationship between specific PMN subtypes and corresponding classes of interneurons. For instance, MiP motoneurons seem to be closely-related to V2a interneurons; in the absence of either Nkx6 or Mnx proteins, MiPs form hybrids that have molecular and morphological characteristics of V2a interneurons [5], [6]. On the other hand, CaP motoneurons and VeLD interneurons seem to be closely-related. In the absence of either Islet or Met proteins, CaPs form hybrids with molecular and morphological characteristics of VeLD interneurons [4], [52]. Additionally, CaP and VeLD also arise from the same progenitor domain, and can even be siblings [23]. Therefore, the initial expression of *inab* in CaP/VaP and VeLD underscores the close relationship between these neurons.

We also demonstrated that *inab* is expressed in hybrid cells that have characteristics of both MiP and CaP motoneurons. We previously showed that in the combined absence of Mnx1, Mnx2a, and Mnx2b proteins, MiPs can acquire characteristics of both CaP motoneurons and V2a interneurons [6]. We were previously able to verify this MiP-CaP hybrid fate by only observing axon morphology, because there are few molecular markers that distinguish PMN subtypes. However, because we now know that *inab* is normally expressed in CaPs but not MiPs, we were able to use this marker to confirm that MiP-CaP hybrids co-express CaP markers as well as CaP morphology.

### *inab* is required for correct VaP axon branching

In addition to being expressed in such a specific subset of cells, the timing of *inab* expression also suggested that this gene may be involved in directed axon outgrowth or the acquisition of axon morphology. CaP, VaP and VeLD neurons begin extending axons around 16–17 hpf [2], [3], [28]. *inab* is first expressed in these cells at 16 hpf, around the time of axogenesis. *inab* expression is also maintained in CaP and VaP through 24 hpf, by which time the CaP axon has completed extension to the ventral aspect of the muscle segment [2]. The specific temporal expression of *inab* immediately before and during axogenesis in CaP, VaP and VeLD places it in the right position to be involved in the acquisition of axon morphology.

Indeed, we have shown that when *inab* is forcibly misspliced, VaP axons have significantly more processes than they do normally, indicating a role for *inab* in axon morphology of VaP MNs. The mechanism by which this axon morphology phenotype arises is unclear – *inab* may normally prevent VaP from reacting to some cue that promotes axon process formation. It is intriguing that although *inab* mRNA is expressed in CaP, VaP and VeLD, its disruption has an effect on only the VaP axon. It is unclear why this differential effect exists. *inab* may be interacting with as-yet-unknown gene products specific to VaP MNs. We also do not know if there is differential expression of Inab protein. Although a working antibody against Inab does not currently exist, it would be interesting to learn whether Inab protein is differentially expressed in CaP and VaP. Regulation at the protein level could contribute to the differential effect of *inab* missplicing in these primary motoneurons.

Also worth noting is that although VaP axon morphology is changed when *inab* is disrupted, VaPs still typically die by 36 hpf, as they do in controls. This observation suggests that Inab is not involved in mediating VaP survival. This observation also indicates that the supernumerary processes of VaP axons are insufficient to overcome the normal interactions with CaP and muscle pioneer cells that are required to prevent VaP from extending a CaP-like axon and ultimately lead to VaP death [26], [27].

## Supporting Information

Figure S1 RNA samples contain spinal cord tissue. (**A–C**) RT-PCR results confirming that all RNA samples contain spinal cord tissue. “Spinal cord”  =  *dbx1*, “Hindbrain”  =  *krox20*, “somite”  =  *myod*. Wild-type RNA sample contains spinal cord tissue (asterisk, A), as well as some contaminating hindbrain tissue and little-to-no somite tissue (A). *dnrbpja*-injected (“supernumerary”) RNA sample contains spinal cord tissue (asterisk, B), as well as some contaminating hindbrain and somite tissues (B). *smoothened* mutant (“decreased”) RNA sample contains spinal cord tissue (asterisk, C), as well as some contaminating hindbrain tissue (C).
(TIF)

Figure S2 *ccdc85al* and *nr2f1b* are expressed in the zebrafish spinal cord. (**A–B**) Single confocal slices of 24 hpf embryos labeled with riboprobe. *ccdc85al* is expressed in cells just dorsal to the *islet1* ^+^ MNs (A). *nr2f1b* is expressed broadly throughout the spinal cord, including expression in *islet1* ^+^ MNs (circles, B-B″). Scale bar, 10 µm.
(TIF)

Figure S3 *inab* knockdown does not affect ventral motor nerve or VeLD axons. (**A–B**) Z-projections of confocal images of control and *inab* MO-injected *mnx1:GFP* transgenic embryos. At 20 hpf, both ventral CaP axons (arrows, A–B) and descending VeLD axons (arrowheads, A–B) are visible in both control (A) and *inab* MO-injected (B) embryos. Scale bar, 10 µm.
(TIF)

Table S1 Candidate genes, their Sequence IDs, and the magnitude and direction of their abundance in the microarrays.
(DOCX)
