# Supplementary figures and images for: The Role of inab in Axon Morphology of an Identified Zebrafish Motoneuron
Source: PLoS One. 2014 Feb 12;9(2):e88631. doi: 10.1371/journal.pone.0088631 (PMC3922942; doi:10.1371/journal.pone.0088631)

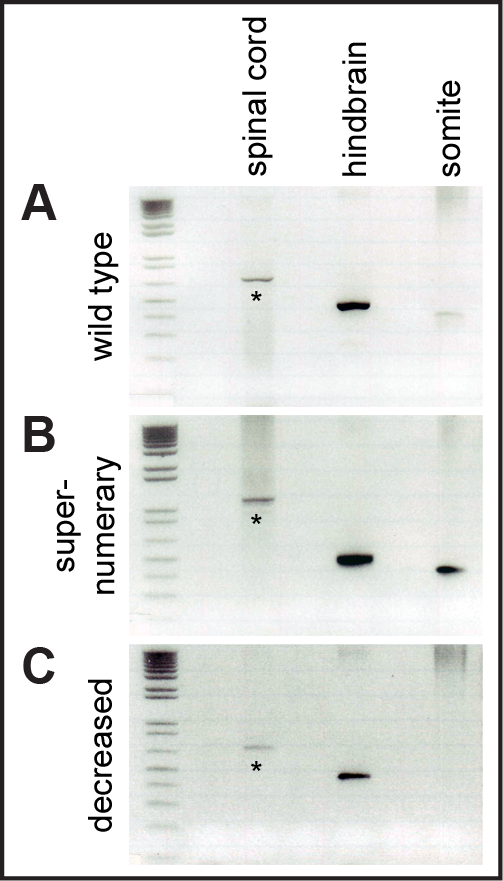

Supplement: Figure S1 — RNA samples contain spinal cord tissue. (A–C) RT-PCR results confirming that all RNA samples contain spinal cord tissue. “Spinal cord” = dbx1, “Hindbrain” = krox20, “somite” = myod. Wild-type RNA sample contains spinal cord tissue (asterisk, A), as well as some contaminating hindbrain tissue and little-to-no somite tissue (A). dnrbpja-injected (“supernumerary”) RNA sample contains spinal cord tissue (asterisk, B), as well as some contaminating hindbrain and somite tissues (B). smoothened mutant (“decreased”) RNA sample contains spinal cord tissue (asterisk, C), as well as some contaminating hindbrain tissue (C). (TIF) [file pone.0088631.s001.tif]

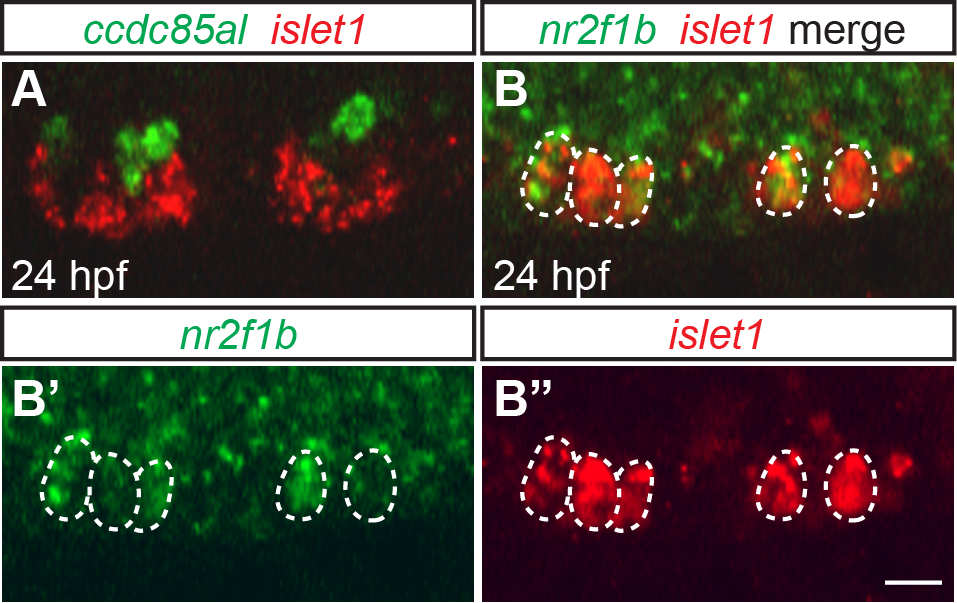

Supplement: Figure S2 — ccdc85al and nr2f1b are expressed in the zebrafish spinal cord. (A–B) Single confocal slices of 24 hpf embryos labeled with riboprobe. ccdc85al is expressed in cells just dorsal to the islet1 + MNs (A). nr2f1b is expressed broadly throughout the spinal cord, including expression in islet1 + MNs (circles, B-B″). Scale bar, 10 µm. (TIF) [file pone.0088631.s002.tif]

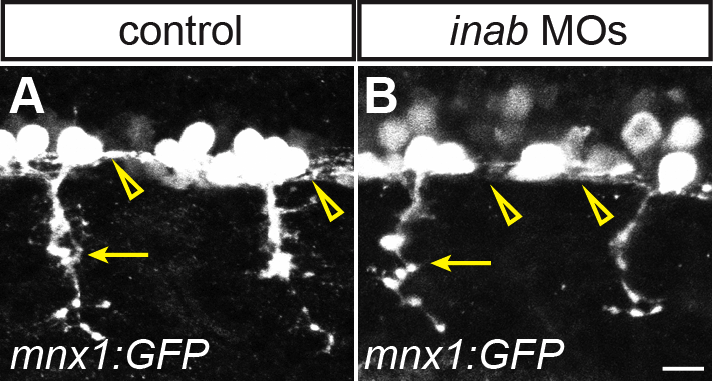

Supplement: Figure S3 — inab knockdown does not affect ventral motor nerve or VeLD axons. (A–B) Z-projections of confocal images of control and inab MO-injected mnx1:GFP transgenic embryos. At 20 hpf, both ventral CaP axons (arrows, A–B) and descending VeLD axons (arrowheads, A–B) are visible in both control (A) and inab MO-injected (B) embryos. Scale bar, 10 µm. (TIF) [file pone.0088631.s003.tif]
