# Supplementary material for: The Role of inab in Axon Morphology of an Identified Zebrafish Motoneuron
Source: PLoS One. 2014 Feb 12;9(2):e88631. doi: 10.1371/journal.pone.0088631 (PMC3922942; doi:10.1371/journal.pone.0088631)
Supplement: Table S1 — Candidate genes, their Sequence IDs, and the magnitude and direction of their abundance in the microarrays. (DOCX) [file pone.0088631.s004.docx]

| gene | Seq_ID | WT to Super-numerary  (fold up) | WT to Decreased  (fold down) |
| --- | --- | --- | --- |
| * *islet1* | ENSDART00000010896 | 1.69 | 2.03 |
| * *nkx6.1* | ENSDART00000029874 | 1.01 | 2.10 |
| * *nkx6.2* | ENSDART00000064702 | 5.39 | 1.32 |
| * *mnx1* | ENSDART00000052236 | 1.18 | 1.13 |
| * *met* | ENSDART00000074359 | 1.35 | 1.21 |
| * *nav1.6* | ENSDART00000022042 | 1.49 | 1.50 |
| * *dla* | ENSDART00000012104 | 1.37 | 1.17 |
| * *neurog1* | ENSDART00000078563 | 1.21 | 1.20 |
| * *nfasca* | ENSDART00000086720 | 1.16 | 1.17 |
| * *mnx2b* | ENSDART00000040368 | 1.24 | 1.67 |
| *mchr1a* | AY161857.1_R | 2.01 | 1.93 |
| *txnipl* | BC055213.1 | 1.84 | 1.28 |
| *ctrb1* | BC055574.1 | 1.56 | 1.52 |
| *slc25a20* | BC062851.1 | 1.66 | 2.22 |
| † ***nr2f1b*** | BC065651.1 | 1.75 | 1.55 |
| *sft2d3* | BC067607.1 | 2.26 | 1.69 |
| *zgc:103764* | BC081526.1 | 1.61 | 1.95 |
| *kng1* | BC083429.1 | 3.46 | 1.64 |
| *zgc:162095* | BC139855.1 | 1.58 | 2.34 |
| *gprc5c* | BC151953.1 | 1.92 | 2.28 |
| *itfg2* | BQ092541_R | 1.52 | 1.61 |
| *scfd2* | BQ262494 | 1.82 | 1.68 |
| *LOC572348* | ENSDART00000011224 | 1.64 | 1.88 |
| *LOC558878* | ENSDART00000020512 | 2.29 | 1.54 |
| *syt9a* | ENSDART00000028693 | 1.65 | 1.94 |
| *ngfrl* | ENSDART00000042249 | 1.80 | 2.30 |
| *LOC100000823* | ENSDART00000048432 | 1.86 | 1.73 |
| *LOC100005686* | ENSDART00000048801 | 1.77 | 1.69 |
| *fgfbp2* | ENSDART00000058466 | 1.54 | 1.81 |
| *ccrl1b* | ENSDART00000058703 | 1.58 | 1.53 |
| *opn3* | ENSDART00000074698 | 1.60 | 1.65 |
| † *zgc:73142* | ENSDART00000075738 | 1.77 | 2.28 |
| *znf385b* | ENSDART00000077091 | 1.77 | 1.51 |
| *LOC562282* | ENSDART00000077215 | 1.57 | 1.55 |
| *cpe* | ENSDART00000078364 | 2.88 | 1.89 |
| *LOC556607* | ENSDART00000078578 | 1.83 | 1.54 |
| *ttc12* | ENSDART00000079405 | 1.51 | 1.63 |
| *chchd3* | ENSDART00000079685 | 1.81 | 1.56 |
| *B8JLJ1_DANRE* | ENSDART00000083790 | 2.12 | 1.94 |
| *LOC557513* | ENSDART00000083883 | 1.52 | 1.68 |
| *ank3* | ENSDART00000088095 | 1.72 | 1.73 |
| *sik2b* | ENSDART00000089953 | 1.67 | 2.08 |
| *LOC560359* | ENSDART00000092578 | 1.58 | 1.76 |
| *trpm4c* | ENSDART00000092687 | 1.51 | 2.15 |
| † *atp2b3a* | ENSDART00000093117 | 1.73 | 1.64 |
| *scospondin* | ENSDART00000097773 | 1.95 | 1.75 |
| *pdlim7* | ENSDART00000098828 | 1.98 | 4.16 |
| *kbtbd10a* | ENDSART00000099787 | 5.14 | 1.79 |
| *pde1a* | ENSDART00000099973 | 1.62 | 2.32 |
| *lrguk* | ENSDART00000101612 | 2.13 | 2.60 |
| *zgc:172158* | ENSDART00000101797 | 3.75 | 2.48 |
| *LOC556841* | ENSDART00000102232 | 2.16 | 1.65 |
| *LOC562421* | ENSDART00000103646 | 1.56 | 1.53 |
| *LOC100151491* | ENSDART00000104884 | 1.78 | 1.64 |
| *fgd4* | ENSDART00000105335 | 2.14 | 1.79 |
| *epdr1* | NM_001002416 | 1.53 | 1.87 |
| *zgc:92620* | NM_001002702 | 4.38 | 1.79 |
| † ***ccdc85al*** | NM_001004570 | 1.76 | 2.06 |
| *ric8a* | NM_001005588 | 1.79 | 1.64 |
| *zgc:103530* | NM_001005991 | 1.81 | 1.54 |
| *zgc:101746* | NM_001006060 | 3.08 | 1.98 |
| *gna13a* | NM_001012243 | 2.69 | 1.80 |
| *ext1c* | NM_001012370 | 3.31 | 1.75 |
| *zgc:92612* | NM_001012372 | 1.82 | 2.13 |
| *zgc:110343* | NM_001013471 | 1.66 | 1.52 |
| *b3galnt2* | NM_001020687 | 1.67 | 1.77 |
| *cnpy2* | NM_001039826 | 1.59 | 1.68 |
| *pdlim3b* | NM_001042718 | 1.83 | 1.72 |
| *kctd7* | NM_001045333 | 1.93 | 1.51 |
| *zgc:153713* | NM_001045350 | 1.72 | 1.92 |
| *zgc:154068* | NM_001045465 | 1.52 | 2.27 |
| *scg2b* | NM_001077748 | 1.61 | 1.98 |
| † *hs2st3l* | NM_001081440 | 2.53 | 1.75 |
| *foxq1l* | NM_001083815 | 1.94 | 2.10 |
| *zgc:162229* | NM_001089522 | 4.63 | 1.85 |
| *zgc:165523* | NM_001098778 | 1.68 | 1.55 |
| *f2r* | OTTDART0000022592 | 1.98 | 1.56 |
| *tlc* | NM_198362 | 2.23 | 1.62 |
| *wwox* | NM_200913 | 2.20 | 1.89 |
| *traf4a* | NM_205762 | 2.13 | 1.56 |
| *nbl1* | NM_207097 | 1.68 | 1.60 |
| *glcea* | NM_212850 | 1.67 | 2.44 |
| *mpzl3* | NM_213169 | 1.64 | 1.53 |
| *zgc:85816* | NM_214747 | 1.71 | 1.55 |
| *phactr2* | OTTDART0000002183 | 1.57 | 2.04 |
| *gpr176* | OTTDART0000006205 | 2.27 | 1.51 |
| † *rxrgb* | OTTDART0000006739 | 1.68 | 1.60 |
| *sema3c* | OTTDART0000009531 | 1.51 | 1.58 |
| *itgb1bp1* | OTTDART0000012669 | 1.55 | 2.56 |
| † *tmtc2* | OTTDART0000012893 | 2.72 | 1.67 |
| *mest* | OTTDART0000014961 | 1.79 | 2.98 |
| *fgf23* | OTTDART0000014983 | 1.63 | 1.58 |
| *sgce* | OTTDART0000018694 | 1.93 | 1.59 |
| *svop* | OTTDART0000019272 | 1.98 | 1.53 |
| *trh1* | OTTDART0000020810 | 1.70 | 1.89 |
| *foxd1* | OTTDART0000021727 | 1.82 | 1.81 |
| *nova1* | OTTDART0000022371 | 2.22 | 1.59 |
| *slc1a3a* | OTTDART0000023123 | 1.86 | 1.72 |
| † *olfm1a* | OTTDART0000023167 | 1.56 | 2.67 |
| *ndrg3a* | OTTDART0000025519 | 1.85 | 2.11 |
| *emx2* | OTTDART0000025974 | 2.27 | 1.50 |
| *gpc3* | OTTDART0000026799 | 1.55 | 1.55 |
| † *fzd8a* | OTTDART0000026880 | 2.00 | 1.54 |
| *tspan15* | OTTDART0000029554 | 2.02 | 1.93 |
| *grm3* | OTTDART0000030339 | 1.92 | 1.58 |
| *arsa* | OTTDART0000030355 | 2.49 | 1.59 |
| *klf11a* | TC265507 | 2.01 | 1.76 |
| † *rap2ip* | ZV700S00000433 | 1.59 | 2.01 |
| *sygr1b* | ZV700S00002236 | 2.29 | 2.32 |
| † ***inab*** | ZV700S00004783 | 1.71 | 2.46 |
| *zgc:110141* | ZV700S00006117 | 2.39 | 1.67 |

**Table S1: Candidate genes, their Sequence IDs, and the magnitude and direction of their abundance in the microarrays.**

* = genes previously known to be involved in motoneuron development

† = candidate genes

**bold** = genes investigated in this paper
